# Draft Genome Sequence of the Bacterium *Lysobacter capsici* X2-3, with a Broad Spectrum of Antimicrobial Activity against Multiple Plant-Pathogenic Microbes

**DOI:** 10.1128/genomeA.00589-15

**Published:** 2015-06-04

**Authors:** Jing-Li Yi, Jing Wang, Qun Li, Zhao-xia Liu, Li Zhang, Ai-Xin Liu, Jin-Feng Yu

**Affiliations:** a Department of Plant Pathology, College of Plant Protection, Shandong Agricultural University, Tai’an, Shandong, China; b Collaborative Innovation Centre for Annually High Yield and High Efficiency Production of Wheat and Corn, Shandong Agricultural University, Tai’an, Shandong, China

## Abstract

*Lysobacter capsici* strain X2-3 was isolated from the wheat rhizosphere in China and exhibits a remarkable capacity to inhibit the growth of multiple pathogens. Here, we report the draft genome sequence of *L. capsici* strain X2-3 in China.

## GENOME ANNOUNCEMENT

The genus *Lysobacter* is composed of 16 species that are typically found in soil and water habitats (1). A striking feature of *Lysobacter* is its lytic activity against other microorganisms, including fungi, bacteria, and nematodes (2). *Lysobacter capsici* is a novel species established in 2008, which was first isolated from the rhizosphere of pepper (3). A few strains of this species that serve as sources of potential biocontrol agents have shown antimicrobial activity against several plant pathogens (4–6). Strain X2-3 was isolated from the wheat rhizosphere and identified as *L. capsici* by 16S rRNA gene sequence analysis (accession no. KP978015). Strain X2-3 showed antimicrobial activities against several plant pathogenic fungi and oomycetes *in vitro*, such as *Rhizoctonia cerealis*, *Rhizoctonia solani*, *Bipolaris sorokiniana*, *Fusarium oxysporum* f. sp. *cucumerinum*, *Verticillium dahliae*, *Colletotrichum gloeosporioides*, *Pythium myriotylum*, *Botrytis cinerea*, *Botryosphaeria ribis*, *Valsa mali*, and *Phytophthora parasitica* var. *nicotianae*. Because of its potential as a biocontrol agent, we describe the draft genome sequence of strain X2-3 to better understand its genetic background.

Genomic DNA was extracted with the cetyltrimethylammonium bromide (CTAB) protocol (7). The genome of *L. capsici* X2-3 was sequenced with massively parallel sequencing (MPS) Illumina technology. A paired-end library with an insert size of 500 bp and a mate pair library with an insert size of 5 kb were constructed. The two libraries were sequenced using an Illumina MiSeq by PE300 strategy and an Illumina HiSeq 2500 by PE100 strategy, respectively. A total of 5,931,492 filtered reads were assembled into 13 contigs (*N*_50_ length, 699,639 bp), with a combined length of 6,126,365 bp. The largest contig was 1,781,518 bp, while the shortest contig was 6,232 bp. Gene prediction of the X2-3 genome assembly was performed with an integrated model, which combined the results generated with GeneMarkS and Heuristic model parameters. A total of 5,117 genes were predicted from the X2-3 genome, in which 4,880 coding sequences were annotated based on BLASTx analysis against the nonredundant protein database. Only 1,861 genes were assigned functions, while 2,019 were considered to code for hypothetical proteins. The G+C content of X2-3 is 66.79%, which is similar to that of *L. capsici* YC5194^T^ (65.4%), *L. capsici* AZ78 (66.43%), and *Lysobacter antibioticus* 13-6 (67.14%) (3, 8, 9).

BLASTn alignments revealed that the *L. capsici* X2-3 genome harbors gene clusters putatively involved in the degradation of chloroalkane, chloroalkene, ethylbenzene, toluene, bisphenol, and polycyclic aromatic hydrocarbon. A number of the genes associated with drug resistance were also identified in the genome*.*

As expected, the *L. capsici* genome contains a number of genes involved in the biosynthesis of nonribosomal peptide siderophores and antibiotics, such as penicillin, cephalosporin, streptomycin, butirosin, neomycin, tetracycline, novobiocin, vancomycin, and ansamycins, which may play important roles in the suppression of plant pathogens. Additionally, the genes coding for lytic enzymes, such as lytic protease, chitinases, glucanases, lipases, and xylanase, were identified in the X2-3 genome. The genetic information of *L. capsici* strain X2-3 provides deep insights into understanding its antimicrobial activity, which is critical for the development of a biological control agent for plant disease management.

### Nucleotide sequence accession numbers. 

This whole-genome shotgun project has been deposited at DDBJ/EMBL/GenBank under the accession no. LBMI00000000. The version described in this paper is version LBMI01000000.
